# Feasibility of Tumor Treating Fields with Pemetrexed and Platinum-Based Chemotherapy for Unresectable Malignant Pleural Mesothelioma: Single-Center, Real-World Data

**DOI:** 10.3390/cancers14082020

**Published:** 2022-04-16

**Authors:** Tugce Kutuk, Haley Appel, Maria Carolina Avendano, Federico Albrecht, Paul Kaywin, Suyen Ramos, Melanie E. Suarez-Murias, Minesh P. Mehta, Rupesh Kotecha

**Affiliations:** 1 Department of Radiation Oncology, Miami Cancer Institute, Baptist Health South Florida, Miami, FL 33176, USA; tugcek@baptisthealth.net (T.K.); haleya@baptisthealth.net (H.A.); mariaave@baptisthealth.net (M.C.A.); suyenra@baptisthealth.net (S.R.); mineshm@baptisthealth.net (M.P.M.); 2 Department of Medical Oncology, Miami Cancer Institute, Baptist Health South Florida, Miami, FL 33176, USA; federicoa@baptisthealth.net (F.A.); paulka@baptisthealth.net (P.K.); melaniebo@baptisthealth.net (M.E.S.-M.); 3 Department of Radiation Oncology, Herbert Wertheim College of Medicine, Florida International University, Miami, FL 33199, USA; 4 Department of Translational Oncology, Herbert Wertheim College of Medicine, Florida International University, Miami, FL 33199, USA

**Keywords:** tumor treating fields (TTFields), malignant pleural mesothelioma, real world experience, compliance, dermatitis, skin adverse events

## Abstract

**Simple Summary:**

Management of malignant pleural mesothelioma (MPM) is challenging as patients frequently present with unresectable disease and the response rates with systemic therapy alone remain low. Given the paucity of effective therapies for MPM, Tumor Treating Fields (TTFields) therapy was made available for use under an FDA-approved Humanitarian Device Exemption (HDE) protocol in 2019, but no real-world data beyond the initial trial have been published to date. We reviewed our retrospective series of five patients diagnosed with MPM and treated with TTFields with pemetrexed and platinum-based chemotherapy. This therapy resulted in a modest disease-stabilization rate with no significant device-related major toxicities. However, we observed universal low-grade skin toxicities related to the device which required medical management and self-discontinuation in 2/5 patients. We also observed lower device usage, compared to the STELLAR trial. Both of these represent opportunities for developing improved management guidelines and efforts to improve patient compliance.

**Abstract:**

Purpose: The objectives of this study were to evaluate the implementation, device usage rates, clinical outcomes, and treatment-related toxicities associated with TTFields and pemetrexed plus platinum-based chemotherapy in patients with unresectable MPM, outside the initial trial results. Methods: Consecutive patients with unresectable MPM were enrolled onto an FDA-required HDE protocol from 2019 to 2021. All patients were treated with a protocol-defined regimen of continuous TTFields (150 kHz) and pemetrexed plus platinum-based chemotherapy. Results: Five patients with unresectable MPM were enrolled. The median number of 4-week TTFields cycles was 5 (range: 2–7 cycles). Median TTFields device usage in the first 3 months was 12.5 h per day (range: 5–16.8 h), representing 52% (21–70%) of the potential daily duration. The median follow-up was 5.4 months (range: 1.1–20.9 months). Treatment-related dermatitis was the only side effect associated with TTFields and was reported as grade 1–2 in all patients; no patient had grade 3+ device-related toxicities. Conclusions: This study represents the first results of real-world implementation of TTFields for MPM. In comparison to the initial clinical trial (STELLAR), compliance rates were lower, although skin-related toxicities appeared similar. Further initiatives and guidelines should be developed to manage treatment-related dermatitis and improve device usage.

## 1. Introduction

Malignant pleural mesothelioma (MPM) is a rare disease linked to asbestos exposure, with a 5-year survival rate of only 10% [1]. Although MPM incidence rates have been declining in the United States, it is estimated that approximately 3000 new cases are diagnosed each year, and the incidence is increasing in the rest of the world, primarily in Asia and Europe [2]. Management of MPM is challenging as patients frequently present with locally advanced disease that is not amenable to surgery, and the majority of chemotherapeutic agents demonstrate low efficacy rates [1,3,4,5]. The combination of pemetrexed and platinum-based chemotherapy is commonly utilized as a standard treatment in MPM [3]. The phase 3 MAPS study found that adding bevacizumab to pemetrexed and platinum-based chemotherapy for first-line unresectable MPM improved overall survival (OS) to 18.8 months compared to 16.1 months with chemotherapy alone. However, bevacizumab was also associated with higher rates of adverse events and is contraindicated in many MPM patients, including those with uncontrolled cardiovascular comorbidities and hypertension [4,6]. Furthermore, this therapeutic combination is not currently widely used as it has yet to receive FDA approval and there are issues with cost and lack of reimbursement. Additionally, dual immune checkpoint inhibitor therapy has been recently approved for MPM patients, although the first-line treatment remains controversial, especially for those with epitheliod histology or PD-L1 negative disease [7].

Tumor Treating Fields (TTFields) are alternating electric fields with low intensity and intermediate frequency, and exhibit antimitotic effects on malignant cells through a polarization effect on the mitotic spindle [8,9]. TTFields are administered non-invasively by a portable electric field generator connected to two pairs of opposing insulated ceramic arrays, placed on the patient’s chest and distributed based on the patient’s gender, size, and tumor extent. The observed antimitotic effects of TTFields include: (1) polar tubulin dimers and septin trimers alignment with the electric field, preventing proper mitosis; (2) accumulation of charged and polar molecules at the cleavage furrow from the dielectrophoretic forces resulting from non-uniform electric fields. These effects lead to abnormal mitosis, impairment of DNA damage repair, the formation of dysfunctional new cells, and ultimately, cellular death [8,9]. Preclinical data have shown that human mesothelioma cells are highly sensitive to TTFields at a frequency of 150 kHz, with a synergistic effect when combined with cytotoxic chemotherapy agents, including cisplatin and pemetrexed [9,10]. The phase 2 STELLAR study evaluated the safety and efficacy of TTFields for the treatment of unresectable MPM concomitantly with pemetrexed and platinum-based chemotherapy [11]. This study reported a high median usage rate with TTFields in the first 3 months (16.3 h per day, which is 68% of the potential daily duration) and a median OS of 18.2 months without an increase in the systemic toxicity associated with chemotherapy, compared to a historic median OS of 12.1 months. The FDA approved TTFields in combination with pemetrexed and a platinum-based chemotherapy via the Human Device Exemption (HDE) pathway for patients with unresectable, locally advanced or metastatic MPM in 2019. Since the FDA approval, our institution has used TTFields with pemetrexed and platinum-based chemotherapy as a treatment option for unresectable, recurrent or metastatic MPM. However, published clinical experience with TTFields in the MPM population outside of the STELLAR trial is lacking. Therefore, the objectives of this study were to evaluate the implementation, compliance rates, clinical outcomes, and treatment-related toxicities associated with TTFields in combination with pemetrexed and platinum-based chemotherapy in patients with unresectable MPM, and compare these with patients on the STELLAR trial. 

## 2. Materials and Methods

Following Institutional Review Board approval, consecutive patients with histologically confirmed unresectable, locally advanced or metastatic MPM who were enrolled onto an FDA-required HDE protocol at a single tertiary care institution from November 2019 to September 2021 were included in this report. Relevant patient data collected from the shared electronic medical record included gender, age, primary tumor histology, race, tumor stage (American Joint Committee on Cancer staging), smoking status, and Eastern Cooperative Oncology Group (ECOG) performance status. In addition, previous treatment details including radiotherapy, chemotherapy, and surgery information were collected. All patients were prescribed TTFields with a usage recommendation of at least 18 h per day applied to the thorax with output parameters of 150 kHz with two sequential, perpendicular field directions at a maximal device output of 1414 milliampere root mean square, using the NovoTTF-100L System (Novocure, Haifa, Israel). 

Treatment with the device was continuous, with breaks allowed for personal needs (e.g., showering, array exchange, etc.). TTFields were initiated on day 1 of treatment concomitantly with chemotherapy. Patients and their caregivers were trained in the use of the device by a trained health care provider and device support specialist. TTFields usage was recorded electronically by the device as average daily use in hours per day, and the information was reviewed and recorded at follow-up visits. Pemetrexed was administered intravenously at a dose of 500 mg/m^2^ together with either cisplatin 75 mg/m^2^ intravenously on day 1 or carboplatin intravenously at a dose of area under the curve (AUC) 5 on day 1. Cycles were to be repeated every 21 days for up to 6 cycles in the absence of disease progression or unacceptable toxicity. In the event of chemotherapy toxicities, dose modifications were employed at the discretion of the treating medical oncologist. Maintenance chemotherapy, including single agent pemetrexed chemotherapy, was prescribed at the discretion of the medical oncologist.

Acute device-related toxicity was defined as any event within 90 days of the treatment and evaluated according to the National Cancer Institute Common Terminology Criteria for Adverse Events, version 5.0 (NCI-CTCAEv5.0). Treatment response was evaluated using Modified Response Evaluation Criteria in Solid Tumors (RECIST) for MPM [12]. Patients were followed every month with updated history, comprehensive physical examination, and evaluation of performance status, adverse events, medication usage, and blood cell count and chemistry evaluation, and every 3 months with computed tomography (CT) of the thorax, or whole-body positron emission tomography (PET) CT. Treatment was continued until radiologic disease progression according to modified RECIST criteria, unacceptable toxicity or clinical deterioration, patient request, or death. In the case of chemotherapy discontinuation due to toxicity or the completion of 6 chemotherapy cycles, TTFields continued until disease progression or unacceptable toxicity. Aside from standard skin care with topical ointment, a high potency topical steroid (clobetasol propionate 0.05% cream) was prescribed if the skin beneath the arrays was inflamed. If the epidermis was breached (skin erosions, ulcers, open sores, punctate lesions, cuts, etc.) an antibiotic ointment (e.g., mupirocin) was prescribed and used in place of the topical corticosteroid. At each array replacement, patients were recommended to shift the placement of the new set of arrays by approximately 0.75 inches (2 cm) compared to the previous layout so that the array discs would be placed between areas of skin irritation. At the subsequent array replacement, the arrays were shifted back to their original location. This shifting of direction was determined by the healthcare provider. If skin toxicity continued despite the above measures, a treatment break was allowed until skin healing was achieved, and a skin barrier prep was recommended prior to additional use of TTFields [13]. 

Descriptive statistics were computed. For continuous variables, the median and range were presented; sample sizes and percentages were presented for categorical variables. OS was defined as the time from TTFields treatment initiation to death or last follow-up. Progression-free survival (PFS) was defined as the time from TTFields treatment initiation to disease progression, death or last follow-up, whichever occurred first. PFS and OS were estimated using the Kaplan–Meier method. Statistical analyses were performed using SPSS, version 27 (SPSS Inc., Chicago, IL, USA). 

## 3. Results

**Figure 1:**
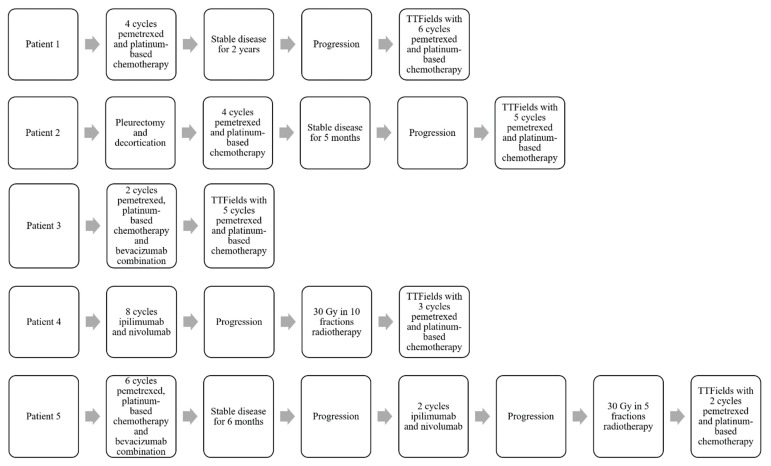
Pre-TTFields treatment details.

Five patients with unresectable MPM were enrolled onto the HDE protocol during the study period. The histologic type was epithelioid in four patients and sarcomatoid in one patient (Table 1). The median age was 69 years (range: 64–84 years) and three patients were male. All patients had good performance statuses (ECOG 0-1). One patient was newly diagnosed and four patients had recurrent disease at initiation of TTFields treatment. Patient #1 was initially diagnosed with stage IB MPM. After multi-disciplinary evaluation, he was not felt to be an optimal candidate for resection given his multiple medical co-morbidities. He received four cycles of pemetrexed and carboplatin and was followed with surveillance imaging for 2 years with stable disease. He then presented with disease progression and was treated with TTFields with pemetrexed and platinum-based chemotherapy. Patient #2 was diagnosed with a stage IIIB MPM for which he received four cycles of pemetrexed and platinum-based chemotherapy after pleurectomy/decortication and after 5 months of observation with stable disease, he presented with disease progression and was treated with TTFields with pemetrexed and platinum-based chemotherapy. Patient #3 was diagnosed with stage IIIA MPM for which she received two cycles of pemetrexed, platinum-based chemotherapy, and bevacizumab. Bevacizumab had to be discontinued due to toxicities, and TTFields with pemetrexed and platinum-based chemotherapy was continued, without chemotherapy treatment interruption. Patient #4 was diagnosed with stage IB sarcomatoid MPM for which he received eight cycles of ipilimumab and nivolumab and subsequently developed disease progression. He was treated with palliative radiation therapy to 30 Gy in 10 fractions to the symptomatic pleural lesions and subsequently initiated treatment with TTFields with pemetrexed and platinum-based chemotherapy. Patient #5 was diagnosed with stage II MPM and given her medical comorbidities, she was not deemed to be a surgical candidate. She started treatment with pemetrexed, platinum-based chemotherapy and bevacizumab (six cycles). She developed recurrence 6 months after her last cycle of chemotherapy, and was started on salvage ipilimumab and nivolumab. Due to disease progression, she was treated with palliative radiation therapy, 30 Gy in 5 fractions, to the symptomatic pleural lesions. After radiotherapy, she initiated treatment with TTFields with pemetrexed and platinum-based chemotherapy. Pre-TTFields treatment details for the five patients with MPM are summarized in Figure 1. 

**Figure 2:**
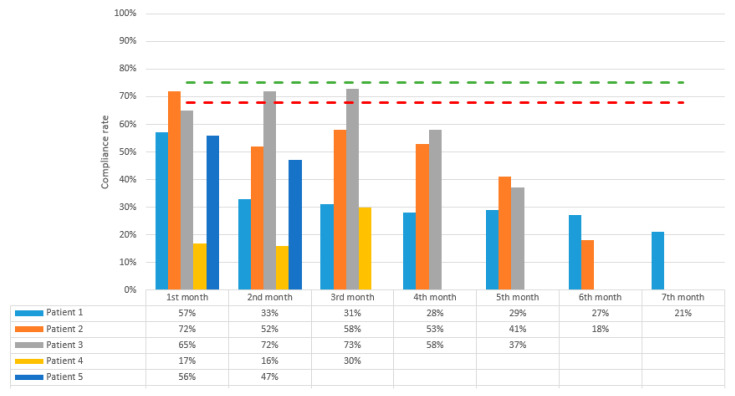
Average daily TTFields device usage percentages by month. The device usage rates recommended by company (green) and recorded by STELLAR study (red) are indicated by the dashed lines.

**Figure 3:**
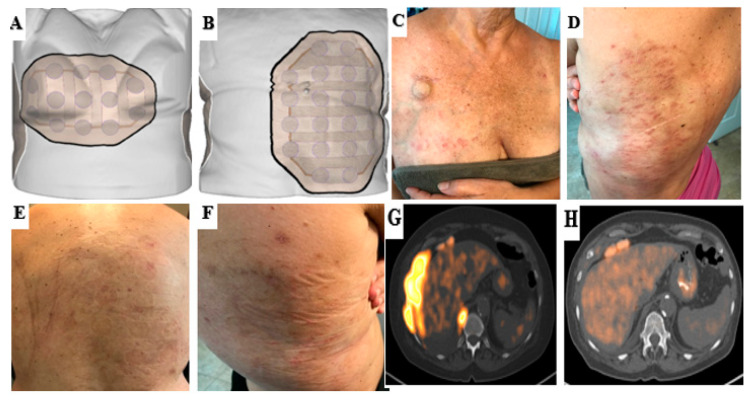
An example case for grade 2 dermatitis associated with TTFields usage. After a few months of use, the patient developed open sores, itching, and dry skin. Mupirocin was recommended for use topically over open sores and a high potency corticosteroid was recommended over the dry itchy skin. A 3-day treatment break from the device was also recommended. (**A**,**B**) A schematic illustration of arrays applied to the front and back thorax region. (**C**,**D**) Grade 2 dermatitis associated with TTFields treatment. (**E**,**F**) After proper skin regimen and treatment break, the open sores healed, and only pruritic skin and discoloration remained. Patient was able to resume treatment with device afterwards with reduction of skin toxicity. (**G**,**H**) After 5 cycles of TTFields with pemetrexed and platinum-based chemotherapy, we observed a near complete response to therapy. There was only minimal thickening and mild hypermetabolic activity remaining along the slip of the diaphragm and at the 9th intercostal space. No adenopathy in the abdomen/pelvis, and no new sites of FDG evidence of metastatic disease were observed in PET/CT.

The median number of pemetrexed and platinum-based chemotherapy cycles was 5 (range 2–6) during TTFields treatment; all patients were treated with the carboplatin and pemetrexed combination. None of the five patients developed any Grade 4+ chemotherapy-related adverse effects (Table 2). The median number of 4-week TTFields cycles was 5 (range 2–7). Median TTFields device usage in the first 3 months was 12.5 h per day (range: 5–16.8 h per day), which is 52% (21–70%) of the potential daily duration (Figure 2). Overall, 2/5 patients developed grade 1 dermatitis (mild symptoms for which topical treatment intervention was indicated and treatment interruption due to skin toxicity of <3 days was required). The remaining three patients developed grade 2 dermatitis (moderate symptoms with interruption of TTFields required for more than 3 days due to skin toxicity). Patients #1, #3, and #4 took a temporary break of at least 3 days because of skin side effects but all resumed treatment after each episode. Patient #3 had to temporarily reduce the use of TTFields due to device-related skin toxicity during month 3 and the addition of a skin barrier prep prior to device placement helped her resume usage with decreased skin toxicity. Similarly, patient #4 had to temporarily reduce the use of TTFields treatment because of device-related skin effects, difficulty with device usage due to hyperhidrosis and peeling of the adhesives of the electrodes. Of note, patient #4 had no prior history of adhesive or latex allergy. Patient #4 developed grade 2 pneumonitis at the 3rd week of the TTFields treatment, which was thought to be associated with prior radiation therapy and immunotherapy given the distribution of his changes. The median TTFields device usage after 3 months decreased to 8.9 h per day (range: 6.2–11.5 h per day), which is 37% (26–48%) of the potential daily duration. Patient #2 discontinued TTFields after the sixth cycle due to distant disease progression. Patients #1 and #3 discontinued TTFields after the fifth and seventh cycles, respectively, as a matter of personal choice. There was no device-related grade 3 or higher skin toxicity (severe or medically significant requiring hospitalization). An example case for grade 2 dermatitis is demonstrated in Figure 3.

The overall median follow-up was 5.4 months (range 1.1–20.9 months) from initiation of TTFields treatment. Four patients were alive at the time of analysis. Patient #2 had a distant progression in gastrohepatic lymph nodes after six cycles of TTFields and five cycles of pemetrexed and platinum-based chemotherapy and died 13 months after the completion of his TTFields treatment due to disease progression. To date, the remaining 4/5 patients continue to have stable disease, and 2/5 patients continue to use the device. The 6-month and 1-year PFS were 80% and 53%, respectively. Median OS and PFS have not been reached yet.

## 4. Discussion

Despite advances in treatment, the prognosis of MPM remains poor. Most patients with MPM are candidates for chemotherapy during the course of their disease, and the combination of platinum with pemetrexed is currently considered the standard first-line approach for patients with disease that is not amenable to resection [1,5]. Given the locally aggressive nature of MPM, the addition of a regional therapy to chemotherapy is very appealing, but the use of radiation therapy is limited by significant pulmonary toxicity [14]. TTFields represent a novel, non-invasive anti-mitotic therapy that permits delivery of a regional therapy to the thorax [15]. Treatment with TTFields in combination with pemetrexed and platinum-based chemotherapy resulted in an OS of 18.2 months, longer than that expected based on prior clinical trials with the same chemotherapy regimen and was well tolerated in unresectable MPM as evaluated in the phase 2 STELLAR study [11]. However, to the best of our knowledge, there is no reported real world clinical experience of TTFields for unresectable MPM to date, outside of the STELLAR data. We reviewed our retrospective series of five patients diagnosed with MPM and treated with TTFields in combination with pemetrexed and platinum-based chemotherapy on an FDA HDE protocol. We found that this combination therapy resulted in modest disease stabilization rate with no significant device-related major toxicities. However, we did observe universal low-grade skin toxicities related to the device which required medical management and self-discontinuation in 2/5 patients. In addition, we also observed lower device usage rates, compared to the STELLAR trial. Both of these represent opportunities for developing improved management guidelines and efforts to improve future patient usage.

In clinical practice, given the limited treatment options, the first-line systemic therapy regimens for unresectable MPM are heterogeneous and include: pemetrexed and platinum-based chemotherapy, pemetrexed alone, platinum-based chemotherapy doublet and bevacizumab, and most recently ipilimumab and nivolumab. However, each of these treatment approaches have advantages and disadvantages leading to heterogeneous patient selection choices in real world clinical practice. Doublet chemotherapy remains the most commonly used approach in the majority of the world, yet the outcomes with this approach alone are poor [5]. Bevacizumab, an anti-VEGF therapy, was shown to increase survival in the first-line setting along with chemotherapy and has been added to the NCCN guideline as a treatment option [16]. However, this therapeutic combination is not currently widely used as it has yet to be given FDA approval in the United States and there are issues with treatment toxicity, cost, and lack of reimbursement in many countries. To this end, we believe that it would be important to pool real world implementation data and treatment outcomes with TTFields in patients treated with different systemic therapy regimens to gain further experience of toxicity and efficacy. Dual immune checkpoint inhibitor therapy (ipilimumab and nivolumab) has also recently been adopted as a standard-of-care first-line treatment for MPM patients [17]; however, chemotherapy can still be considered a first line approach for epitheliod histology and those with a PD-L1 < 1%. Moreover, there is also a potential risk of hyper-progressive disease with dual immune checkpoint inhibitor therapy alone in an unselected MPM population [7]. Since MPM progresses regionally within the thorax, the addition of a local therapy to these systemic therapy regimens remains a very appealing approach to treat this disease. Given the preclinical data demonstrating that TTFields and concurrent anti-PD-1 therapy enhances antitumor immunity [18] combined with the compelling outcomes from the epitheliod subset of the STELLAR trial, we would consider a novel MPM trial in patients with epithelioid histology with TTFields and dual immune checkpoint inhibitor combination a logical next investigational step to optimally address local and systemic disease.

Treatment non-compliance is a major clinical issue for several reasons. Previous studies showed significant improvement in median OS with daily TTFields usage rates of ≥75% versus <75% (for treatment of central nervous system malignancies), establishing the same practice standard for other disease sites [19,20,21]. For MPM, in the phase 2 STELLAR study, Ceresoli et al., reported that the median TTFields device usage in the first 3 months was 16.3 h per day, which is a 68% usage rate [11]. In this study, we observed a median usage of 12.5 h per day (52%) for the first three months. Additionally, unique to our study, we also reported the compliance rates after the 3-month period, which were even lower at 8.9 h per day (37% of the potential daily duration). The significantly lower compliance in our series is likely multi-factorial in nature, including climate differences (our institute is located in a hot and humid climate zone), patient age, lack of enrollment in a strict clinical trial such as STELLAR, and also potentially related to 4/5 having recurrent disease at initiation of TTFields (vs. upfront disease, as was the case in the STELLAR trial) and therefore a more fragile patient population. Similar to our study, Rivera and colleagues reported a median 14 h per day (58%) usage rate, which is 78% of the recommended 18 h/day, with TTFields with gemcitabine alone and a median 12.2 h per day (51%) usage rate, which is 68% of the recommended 18 h per day, in the TTFields plus gemcitabine and nab-paclitaxel group in their study of patients with pancreatic cancer [22]. Vergote et al. also reported a median 14 h per day usage rate (58%, 77% of the recommended 18 h/day) with TTFields during the first 3 months in their study of recurrent ovarian cancer patients [23]. Usage rates and skin toxicity rates for presented or published TTFields clinical trials are summarized in Table 3. Ultimately, preclinical and clinical studies will be needed to define the optimally relevant device usage windows for each disease and biofeedback efforts to track these rates (i.e., connectivity to a patient’s electronic devices for immediate and short-term feedback) may be necessary to improve these rates.

The most common side effect associated with TTFields observed in all five patients in this series was skin irritation beneath the arrays. For this reason, it is important to monitor not only the patient’s clinical status and treatment-related toxicities from chemotherapy, but also their skin care regimens. Proper skin care can help reduce the risk of developing skin irritation while using TTFields and may help increase monthly usage [13]. In this study, we observed grade 1–2 skin side effects in all patients. All dermatologic adverse events were local and mild or moderate in nature. They were primarily managed with topical interventions and relocation (shifting) of the arrays. Previous studies have reported a wide range of skin toxicity rates (16–90%) with TTFields, but a majority of these were mild and moderate side effects with low discontinuation rates [11,22,23,24,25]. Multiple patient, tumor, and device-related factors can affect the risk of developing skin side effects in patients treated with TTFields. Potential patient-related factors include the skin condition of the patient (growth of hair and hygiene of the skin, physical activities, life-style, etc.), frequent array changes, and skin trauma due to inappropriate or vigorous array removal. Patients who previously developed contact dermatitis to any materials used on the arrays, have hyperhidrosis, have received radiotherapy to superficial chest wall regions, or symptomatic tract recurrences might also have a higher risk of skin toxicity [13,27]. In our cohort, two patients had prior radiotherapy to the chest wall for symptomatic chest wall pain, and although they did not develop radiation-related dermatitis prior to TTFields treatment, this could have increased the risk for skin toxicity. In the STELLAR study, no patient received radiotherapy prior to TTFields treatment. Other factors which can increase the risk of skin toxicity include systemic therapies (i.e., bevacizumab-associated wound healing complications) or usage of high dose systemic corticosteroids which may be observed as TTFields therapy permeates into clinical practice and patients receive differing systemic therapies [13,27]. All patients in this study were treated with a protocol-defined platinum and pemetrexed combination; however, recurrent patients had also received different agents previously including bevacizumab and immunotherapies. Not to be discounted are product-related factors, including the arrays (constant pressure from discs or friction from adhesive tape), thermal reaction (heat generated from ceramic discs), chemical irritation (hydrogel, adhesive), and moisture (lack of breathability of array materials, ambient temperature and humidity, clothing). These product-related factors were encountered by all patients in this study and even if not associated with high-grade toxicity, ultimately likely contributed to the discontinuation of the device in two patients.

**Figure 4:**
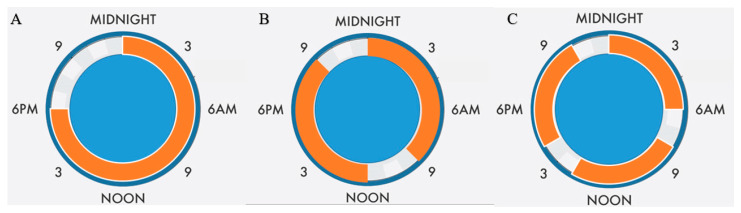
Examples for suggested fractionation schedules for TTFields treatment. In scenario (**A**), 18 h daily usage continuously and 6 h off time is illustrated. In scenario (**B**), 9 h usage and 3 h break time twice daily is illustrated. In scenario (**C**), 6 h usage and 2 h break times three times daily is illustrated.

In oncology, the analysis of real-world data to answer clinical questions that cannot be directly or completely answered using data from clinical trials has gained interest in recent years [28]. It is clear that data obtained from patient records can produce valuable insights into treatments and outcomes in daily oncology practice. Real-world studies can also give information about the compliance or adherence to treatment that can be conditioned by toxicity but also by feasibility of the regimen and patients’ motivation. Our study reflects the compliance rates in real world implementation on the use of TTFields in MPM patients and represents an opportunity for improvement. Effective skin care strategies and management guidelines may improve compliance with TTFields therapy [26]. One of the other strategies might be a fractionation approach of TTFields delivery which might allow time for the recovery of normal epithelial cells. An example of a possible fractionation schedule is demonstrated in Figure 4. 

Our study has several limitations. First, given the retrospective nature of the study, prior treatments were not standardized with respect to chemotherapy and radiotherapy regimen. Second, a limited number of patients with heterogeneous clinical stages were enrolled onto this HDE protocol over the last 2 years due to the rarity of the MPM diagnosis and indications for TTFields treatment. Third, our study has a short follow up interval limiting long-term side effects evaluation or survival estimates. We do report the acute toxicity experience and TTFields patient usage rates in our study, however longer follow up is needed to draw conclusions on long-term safety and efficacy. Fourth, because of the retrospective design of our study, we did not have patient-reported outcomes or quality of life metrics recorded for the patients. 

## 5. Conclusions

In conclusion, this study represents the first analysis of real-world implementation of TTFields with pemetrexed and platinum-based chemotherapy in MPM treatment to date. We showed that this treatment combination is a feasible treatment strategy for appropriately selected patients. Further strategies are required to increase treatment usage rates since the real world usage was lower than the suggested daily usage and help to manage device-related skin adverse effects. 

## Figures and Tables

**Table 1:** Patient and disease characteristics.

	Age (Years)	Gender	Race	Primary Histology	ECOG Status	Smoking Status	AJCC 8th Edition Stage	Disease Status at the Time of TTFields Treatment
Patient 1	84	Male	White Hispanic	Epithelioid	1	Non-smoker	T2N0M0 (Stage IB)	Recurrent disease
Patient 2	69	Male	White Caucasian	Epithelioid	0	Non-smoker	T4N2M0 (Stage IIIB)	Recurrent disease
Patient 3	67	Female	White Caucasian	Epithelioid	1	Former smoker (20 pack/year)	T3N1M0 (Stage IIIA)	Newly diagnosed
Patient 4	75	Male	White Hispanic	Sarcomatoid	1	Non-smoker	T2N0M0 (Stage IB)	Recurrent disease
Patient 5	64	Female	White Hispanic	Epithelioid	1	Former smoker (10 pack/year)	T2N1M0 (Stage II)	Recurrent disease

**Table 2:** Side effects for TTFields with pemetrexed and platinum-based chemotherapy regimen.

	Grade 1	Grade 2	Grade 3	Grade 4–5
Dermatitis	2 (40%)	3 (60%)	0	0
Neutropenia	0	1 (20%)	1 (20%)	0
Anemia	3 (60%)	2 (40%)	0	0
Fatigue	3 (60%)	2 (40%)	0	0
Oliguria	0	1 (20%)	0	0
Pain	0	1 (20%)	0	0
Renal toxicity	0	1 (20%)	0	0
Pneumonitis	0	1 (20%)	0	0

**Table 3:** Summary of the skin adverse events and usage rates of published clinical trials of TTFields in various disease sites.

	Diagnosis	N	Patients with Skin Side Effect (N, %)	Patients with Grade 3 Skin Side Effects (N, %)	TTFields Device Usage Rate
Stupp et al. (EF-11) [24]	Recurrent GBM	116	19 (16%)	0	86%
Stupp et al. (EF-14) [25]	Newly diagnosed GBM	456	237 (52%)	9 (2%)	75% of patients used ≥18 h/d (first 3 months of treatment)
Ceresoli et al. (STELLAR) [11]	Unresectable MPM	80	57 (71%)	4 (5%)	68% (first 3 months)
Rivera et al. (PANOVA) [22]	Locally advanced or metastatic pancreas adenocarcinoma	40	21 (53%)	7 (18%)	58% (TTFields with gemcitabine alone) 51% (TTFields plus gemcitabine and nab-paclitaxel)
Vergote et al. (INNOVATE) [23]	Recurrent ovarian carcinoma	31	28 (90%)	2 (6%)	58%
Pless et al. [26]	Inoperable stage III and stage IV NSCLC	42	18 (43%)	1 (2%)	47%

GBM: glioblastoma, MPM: malignant pleural mesothelioma, NSCLC: non-small cell lung cancer.

## Data Availability

Research data are stored in an institutional repository and will be shared upon request to the corresponding author.
